# Supplementary material for: Effectiveness of a videoconferencing group-based dyad acceptance and commitment therapy on the quality of life of chronic heart failure patients and their family caregivers: A study protocol for a randomized controlled trial
Source: PLoS One. 2024 Apr 18;19(4):e0298178. doi: 10.1371/journal.pone.0298178 (PMC11025806; doi:10.1371/journal.pone.0298178)
Supplement: S1 Protocol — (DOCX) [file pone.0298178.s002.docx]

**Effectiveness of a group-based acceptance and commitment therapy on quality of life in patients with Chronic heart failure and their family caregivers: A randomized controlled trial**

| Principle investigator | Xuelin ZHANG |
| --- | --- |
| Department | Cardiovascular department, Taihe Hospital |
| Study Period: | May 2022 - December 2023 |
| Version Number | V 3.0 |
| Version Date | May 25, 2022 |

# Introduction

Chronic Heart Failure (CHF) is a cardiovascular condition characterized by progressively debilitating symptoms and frequent life-threatening exacerbations (1, 2), affecting 26 million individuals globally, along with their families (2) . Patients with CHF are confronted with numerous physical burdens, arduous medical treatments, and profound lifestyle adjustments (3-5) . They also experience uncertainty in illness, fear of sudden death, anxiety, depression, and non-adherence to recommended self-care activities for CHF. These factors are often intertwined and linked to compromised health-related quality of life (HRQOL) for CHF patients (6-10). Family caregivers (FCs) are essential in providing emotional and practical support to CHF patients to improve health outcomes (11, 12). However, after the diagnosis of CHF, the majority of FCs (94%) experience occupational, physical, psychological, and social burdens (13), with nearly 86.7% of FCs indicating high levels of caregiving burden (14). A considerable proportion of FCs report depressive symptoms (23% to 47%) (15) and anxiety (50%) (16). The psychological states of both caregivers and patients are linked to the HRQOL of FCs (17-19). During the process of managing CHF, family conflicts, relationship issues, and communication problems have been reported as consequences of psychological distress in families living with CHF(20, 21). FCs’ HRQOL is as impaired as that of CHF patients (17).

Clinical guidelines for managing CHF emphasize the importance of incorporating FCs in the CHF care process (22, 23). Research has indicated that dyadic interventions, which involve both patients and FCs, are promising in reducing hospital readmission rates and improving the health-related quality of life (HRQOL) for patients with chronic illness, and better mental health outcomes for FCs by facilitating disease self-management (24, 25). However, non-pharmacological aspects when combined with the efforts of FCs in the context of CHF home care have been understudied (26). In a recent systematic review (26), three experimental studies provided non-pharmacological support to optimize HRQOL and related outcomes for CHF patients and their FCs (27-29). The studies mainly employed strategies such as providing education and psychological support to patients and caregivers after discharge, but the impact on HRQOL and other outcomes remained inconclusive. Moreover, the psychological component employed including cognitive-behavioral therapy (CBT), and motivation enhancement, may not be sufficient to promote CHF self-management (30), and may even result in patients avoiding recommended self-management behaviors (31), potentially leading to poor health outcomes.

Acceptance and commitment therapy (ACT) is a therapy that focuses on the context in which thoughts and emotions occur, with the goal of changing the functions of problematic behavior rather than altering cognitive content (32). By fostering acceptance of private experiences and decreasing fusion with negative thoughts, ACT can promote HRQOL by allowing individuals to behave in line with their values and lead a meaningful life (32). However, empirical research on the use of ACT to improve HRQOL for CHF patients and their FCs is scarce. While recent systematic reviews have shown promising effects of ACT on the HRQOL of patients with chronic illness (33) and FCs (34), most studies have been conducted at the individual level with less attention paid to the circumstances of context. Two pilot studies conducted among patients with advanced cancer have shown early support for the use of a remote ACT intervention for patient-FC dyads, but with no statistically significant differences found in HRQOL, usage of health services, and caregiver distress when compared to the usual care (35, 36). Methodological shortcomings, including the exclusion of relational measures, the use of a telephone-based modality, and small sample sizes, may limit the validity of the studies. Previous studies indicated that establishing a group context and using commonly accessible videoconferencing platforms (37, 38)may improve the effects of an ACT intervention.

Given the shortcomings and strengths of these interventions and considering the availability of patients and their FCs and the potential positive effects of group-based interventions, what might be needed is an online, videoconferencing, group-based intervention using ACT for patients and FCs. This protocol of study aims to bridge the gap by testing the effectiveness of group-based dyad-orientated ACT for patients and their family caregivers.

**2 Methods**

**Objectives**

This study is proposed with the objective of evaluating the effectiveness of a dyad ACT-based intervention delivered via smartphone for CHF patients and their FCs compared with CHF education only at a 3-month follow-up on: (1) Patient and FC outcomes: HRQOL (primary outcome), perceived health status, anxiety, depressive symptoms; perceived quality of the relationship, psychological flexibility, self-compassion, and perspective-taking; (2) Patient-only outcomes: Hospital service utilization; CHF self-care behavior; and (3) Caregiver-only outcome: Caregiving burden.

**Study design**

The proposed study is a two-armed, parallel-group, equivalence randomized controlled trial (RCT). Dyads of CHF patients and their FCs will be randomized at a 1:1 ratio to either the intervention group, which will receive an ACT-based intervention, or the control group, which will receive only CHF education.

**Study setting**

## The participants will be recruited from the Department of Cardiology at Taihe Hospital in Shiyan City, China. The hospital is the largest public hospital in Shiyan city, which as a total population is 3,209,004, according to the 2020 census (39). The Department of Cardiology is responsible for the prevention, treatment, and rehabilitation of residents with cardiovascular disease, including CHF.

## Annually, about 3,600 patients are hospitalized with a diagnosis of CHF. After CHF patients are discharged from the hospital, they will be expected to visit the heart failure clinic to review their health condition every one to three months. They will also receive the usual care, which is in line with national and local clinical CHF guidelines in mainland China (23). The usual care includes follow-up telephone calls to provide educational support based on the inquiries of patients and to remind patients of the necessity of undergoing a medical check-up conducted by a physician one week after discharge and by a clinical nurse one month after discharge.

# Eligibility

## Participants of the present study involve both patients with CHF and their corresponding primary FCs. CHF patients will be eligible if they are 18 years of age or older, have been clinically diagnosed with CHF (40) as indicated in their electronic medical records, and with a New York Heart Association (NYHA) Classification of I to III as confirmed by a physician in this study, have been hospitalized in the past one year. CHF patients will be asked to nominate their primary FC, who would be the one with the highest average number of contact hours among their family members, to join the program. FCs should have aided patients with care needs for a minimum of 14 hours per week consistently for at least three months. FCs should be 18 years of age or older, and they can be patients’ spouse, parent, or adult child, but not a paid caregiver or other relative. In addition, at least one member of the patient-caregiver dyad will be required to have a smartphone installed with the WeChat app and a data plan. Both members should understand and communicate in Chinese, as well as be able to access Tencent VooV Meeting via smartphone to attend the sessions.

## Dyads will be excluded from the program if either member has a cognitive impairment screened with a score of 0-2 on the Mini-Cog scale (41) and a documented medical history of psychiatric illness, dementia, or a life-threatening illness, such as severe pulmonary disease or end-stage renal failure. The dyads of patients who live in nursing homes will also be excluded.

## Patients and their FCs will be required to participate in the group sessions on a dyad basis. During the intervention period, if either member has been hospitalized, sessions will be suspended during the period of hospitalization and will resume after she/he is discharged to go home.

# Sample size calculation

## A prior power analysis was performed to estimate the sample size needed in this study using G*Power. Previous studies in CHF have shown that mean differences in HRQOL between patients who received a psychosocial intervention and the usual care ranged from 0.11 to 0.68 (42). For ACT interventions targeting HRQOL, a recent review meta-analysis reported a mean effect of 0.48 for the clinical and non-clinical population, ranging from 0.37 to 1.55 when those who received the ACT intervention were compared to the active control, not including CBT (43). Thus, an estimated effect size of 0.50 was adopted across the above reviews. Targeting that effect size at a power of 0.8 with a prior power calculation, a minimal sample size of 128 is required to detect the difference between the ACT group and CE groups. Considering a weighted dropout rate of 16% for those participating in an ACT intervention indicated in a recent meta-analysis (44), and given group-setting and patient-caregiver dyadic participant requirements for the interventions, the total sample size was increased to 160 patient-FC dyads, accounting for an anticipated dropout rate of 20%.

# Randomization and blinding

## In this study, allocations with a ratio of 1:1 will be performed through an online randomization program (<https://www.randomizer.org/>) by an independent researcher, who will be excluded from any other activity throughout the intervention. The unit of randomization is the patient-FC dyad. The randomization list will be stratified by the type of relationship between the patients and caregivers (e.g., spousal relationship vs. non-spousal relationship) with a block size of 4 or 6. The allocation sequence will be printed and sealed separately in opaque envelopes by the randomizer.

## The staff nurses in the study hospital will enroll dyads of patients and caregivers. After the baseline assessment is completed, the dyad’s registration data will be given to the research coordinator. The research coordinator will open the envelope and then enter the data into the database and assign dyads to interventions. The participants will be informed of their allocation status via smartphone when the research coordinator plans to schedule the first session.

## The research assistants serving as outcome assessors in this study will be blinded to the group allocations; they will be unable to access the participants' intervention conditions during the program. During the follow-up assessments, the outcome assessors will remind the participants to remain blind to their group assignment throughout the study and ask them to conceal their status during the outcome assessments. If this blinding is breached for any reason, another research assistant who is unaware of the group condition of the dyads will take over the phonic assessment. Participating dyads and interventionists will not be blinded because of the nature of the psychoeducational intervention.

# Interventions

Participating dyads will be allocated to either the intervention group or the control group. All of them will receive four identical weekly sessions via the Tencent VooV meeting over four consecutive weeks in addition to the usual care provided by the department of cardiology. Each session will include four to eight dyads and last approximately two hours, and will be led by a registered nurse(s) with technical support from two research assistants throughout the sessions. Participants will receive relevant materials and handouts prior to the first session, and they will be instructed to retain these materials and handouts throughout the duration of the study and beyond. The online sessions will be scheduled on weekday evenings and weekend afternoons to accommodate typical family events and work schedules. During the period of the interventions, WeChat voice calls or text message reminders will be sent to the dyads before each session to improve participant retention and support engagement in the program.

# Intervention group: ACT-based intervention (ACT group)

Each session in the intervention group will consist of group ACT (1.5 hours) and brief CHF education (0.5 hours) conducted by two trained registered nurses as facilitators. The ACT intervention covers six interrelated core processes to produce psychological flexibility by using metaphor illustrations, experiential exercises, and guided mindfulness exercises, while the contents of the brief CHF education only include information on CHF self-care. Prior to the session, the participant will receive an additional packet supporting the practice of ACT exercises in the sessions, which will include black and white cards, whiteboard markers, two small packages of raisins, and one towel, in addition to one set of CHF self-care materials. The handouts for this group consist of information on CHF self-care, ACT skills, and homework assignments making changes to the ACT processes targeted in each session. Participants will be asked to maintain the confidentiality of the intervention content and avoid discussing it with participants in the control group to prevent contamination.

**ACT-based intervention development**

The first version of the ACT intervention protocol was informed by the ACT classic therapist manual (32, 45-51) and literature on the experiences of CHF patients and FCs (52-56), previous ACT trials with chronic illness patients and FCs (57-61), and an ACT intervention at the dyadic level (36, 62-64). The intervention protocol (Version 1) was initially evaluated in a 4-week uncontrolled clinical trial through an in-person format (𝑁 = 5). This led to some adaptations of the ACT intervention in relation to the dyads. Afterward, the intervention protocol was refined and adopted in an online videoconference format (Version 2). The intervention protocol (Version 2) was retested in a 4-week pilot RCT via smartphone without a follow-up assessment (𝑁=14); the findings indicated that further refinements were required of some experiential exercises, and they informed the revision of the intervention protocol (Version 3) before the commencement of its implementation in this RCT. All versions were finalized by the research team and informed by feedback from the participants and facilitators, the facilitators’ direct observations during the delivery of the intervention, and input from the ACT expert. Any disagreements were discussed with the chief supervisor and resolved by consensus.

The four-session group-based ACT intervention includes exploring participant’s control agendas, identifying their values, exploring their thoughts and feelings, and finding ways to take committed actions in line with the personal values that they hold. The first ACT sessions are aimed principally at exploring previous control-based strategies to attempt to cope with private experiences and the workability of control when pursuing meaningful lives. The following session starts with a review of the processes in which the participants were instructed in the previous session and the between-session assignments. From the second session, the facilitators will focus on increasing connection with life values and commitment to values-based actions. Individual values will be identified, while alternative behavior related to the participants’ values will be clarified. Patient-FC dyads will have the opportunity to share their values and alternative value-based actions to manage CHF situation with each other. Potential cognitive and emotional barriers in the context of home care to adopt alternative behavior and preserve value-driven long-term action will be explored and addressed via illustrations using metaphors and by practicing experiential exercises. Participants will be encouraged to be more accepting and compassionate, detach themselves from self-conceptualizing, take a flexible perspective, and develop flexible attention. Throughout the program, facilitators will facilitate dyadic sharing and reflect on their experience, which in turn will promote the establishment of supportive relationship bonds.

**Facilitator training and fidelity checks**

Two registered nurses will deliver the ACT intervention to the patient-FC dyads. The primary facilitator is also a doctoral student in nursing who has completed a total of 7 days of ACT workshops and a guided online 21-day ACT action camp led by ACT experts worldwide and in China. The co-facilitator is a registered nurse at the local hospital who holds a bachelor's degree in nursing and has received two days of introductory-level training in ACT led by ACT experts in China. Both of them have at least three years of experience working with cardiac inpatients and have co-facilitated the delivery of about eight sessions of a group-based ACT intervention for patient-FC dyads. The facilitators' prior training will be supplemented through role-play practices using the finalized version of the intervention protocol for this study before the commencement of this study.

To monitor the facilitators' competence and fidelity to the contents of the intervention protocol, each group session will be video-recorded with participant’s consent and reviewed independently by facilitators immediately after the session by using the ACT core competency self-rating form (65). Inter-rater agreement will be established before the implementation, while the intra-class correlation (2,1) for each session will be calculated. Feedback on fidelity will be discussed during an in-person meeting after each session. Besides peer coaching, regular bi-weekly supervision and timely supervision will be provided by an experienced ACT researcher to ensure adherence to the intervention protocol throughout the program. The doctoral degree student facilitator and her supervisor will together review the difficulties encountered during the sessions. If any area for improvement is identified, the primary facilitator will provide a remedy before the next session.

# Control group: CHF education (CE group)

CHF education only as an active control was chosen because knowledge of CHF is a foundational and essential component to empower patients with CHF and their FCs to manage CHF in the family context (66).

The sessions for the CHF education control will be delivered by one registered nurse who holds a bachelor's degree in nursing and has at least three years of experience working with cardiac inpatients. Each 2-hour session includes a review of the previous session, didactic education, and a Q&A section to evaluate the participants’ understanding of the key concepts. During the CHF didactic education, participating dyads will receive CHF education information provided by the staff nurse. The contents of the CHF education have been mapped based on the latest national clinical practice guideline for CHF (23). Apart from information on CHF self-care, the control group will receive additional knowledge on CHF, involving the definition of CHF, epidemiology, diagnosis, comorbidity, and treatment.

Prior to the sessions, dyads will be provided with one set of CHF self-care materials, including two salt spoons, one portable pill box, a scale and cup measurement, and a hard copy of handouts. The handouts for the control group consist of information on CHF self-care and homework assignments using well-established questionnaires, such as the heart failure knowledge test (67), the Dutch Heart Failure Knowledge Scale (68), and the heart failure specific health literacy scale (69) . Table 1 lists a summary of the ACT group and CE group for CHF patients and their FCs.

**Table 1.** Summary of the ACT group and CE group sessions

| **Session** | **ACT group (Intervention group)** | **CE group (Control group)** |
| --- | --- | --- |
| Week 1  Session 1 | - Introduction to the sessions, guidelines, and group expectations - Introducing and practicing mindfulness when eating raisins - Exploring the control agenda: identifying issues, the normalcy of psychological suffering, unworkability of the control-based strategies by practicing guided mindfulness to revisit a challenging moment during the CHF management process in the family context - Introducing the paradoxical effects of control-based strategies and acceptance as an alternative by using the metaphor of quicksand - Providing an overview of CHF self-management and symptom monitoring - Homework assignment: to identify challenging moments and be aware of inner events | - Introduction to the sessions - Providing information on CHF facts and self-management - Definition of heart failure - Terminology of heart failure - Heart failure epidemiology and prognosis - Key steps in the diagnosis of CHF - Importance of CHF self-management* - Importance and strategies of monitoring symptoms* - Reviewing the main concept taught in this session based on the following questions   What are your symptoms of CHF?  Why is it critical to check the signs and symptoms of CHF every day?  How do you plan to watch for your signs and symptoms of CHF every day?   - Homework assignment: adopted the heart failure knowledge test (67) |
| Week 2  Session 2 | - Practicing mindfulness when listening - Reviewing the previous session - Clarifying personal values with a “value in trash” exercise and exploring how individuals may choose to respond to negative thoughts/feelings about managing CHF in a value-consistent manner - Exploring the unworkability of attempts to avoid or control negative internal experiences (e.g., relationships, thoughts, feelings) and how these efforts lead to value-inconsistent actions and impaired quality of life by illustrating the metaphor of “the passengers on the bus” - Providing information on recognizing the most common cardiac medication regime, the correct use and possible side effects, and the importance of medication adherence and possible solutions - Homework assignment: identify an alternative behavior they are willing to perform that would help them move toward their core values | - Reviewing the previous session - Providing information on CHF pharmacological treatment and medication adherence - Pharmacological treatment for CHF - Correct use and possible side effects* - Importance of medication adherence and possible solutions* - Reviewing the main concept taught in this session based on the following questions   What is the name of your ordered medicine?  Why is it important to take your medicine every day?  How will you remember to take your water pill every day?   - Homework assignment: adopted the Dutch Heart Failure Knowledge Scale (68) |
| Week 3  Session 3 | - Practicing mindfulness when breathing - Reviewing the previous session - Identifying an alternative behavior they are willing to perform that would help them move towards their core values - Exploring possible inner barriers (e.g., cognitive or emotional) to taking these committed actions - Promoting detachment from unhelpful thoughts, feelings, and prior relationship experience about CHF and CHF management in the context of home care by engaging in defusion exercises, including “thoughts on the cards” and a “tug of war” - Providing information on the importance of fluids and dietary management, a low-sodium diet, and tips on selecting food every day - Homework assignment: establish an action plan for an identified alternative behavior, implement the specific behavior, and be mindful of the response of family members. | - Reviewing the previous session - Providing information on CHF treatment, dietary and fluid management - Cardiovascular and non-cardiovascular comorbidities - Oxygen therapy and ventilatory therapy - Mechanical circulatory support - Heart transplantation and renal replacement - Importance of fluids and dietary management* - Low-sodium diet* - Tips on selecting food every day* - Reviewing the main concepts taught in this session based on the following questions:   What foods should you avoid?  Why is it essential to reduce salt intake?  How do you plan to decrease the sodium in your daily diet?   - Homework assignment: adopted the patient knowledge questionnaire on heart failure (69) |
| Week 4  Session 4 | - Practicing mindfulness in a brief body scan exercise - Reviewing the previous session - Clarifying helpful steps to take for patients and their FCs when painful experiences are aroused - Allowing participants to extend self-understanding and self-compassion to their family members by practicing guided mindfulness and revisiting their past experiences of conflict in the context of family - Summarizing the ACT skills taught in the session - Identifying some possible future challenges and knowing how these skills may be useful - Providing information on CHF physical activities, and cigarette and alcohol consumption | - Reviewing the topics discussed in previous sessions - Providing information on CHF physical activities, and cigarette and alcohol consumption - Advantages and disadvantages of regular physical exercise* - Common principles during exercise* - Monitoring pulse rate to adjust the intensity of exercise* - Recognizing and responding to possible cardiac symptoms during the exercises* - The benefit of smoking cessation and common cessation methods * - Abstaining from excessive alcohol intake* - Reviewing the main concepts taught in this session, based on the following questions   How much of a weight gain should you report to your doctor?  Why is it important to weigh yourself daily?  How can you remember to weigh yourself daily? |

Note: ACT: Acceptance and commitment therapy; CHF: Chronic heart failure; HRQOL: Health-related quality of life. * The content is similar to that in the corresponding session in the intervention group.

# Outcomes

The characteristics of the participants will be gathered at baseline before the intervention, including: (1) their sociodemographic data, such as their age, gender, educational attainment, employment, and relationship with each other; and (2) their clinical data, such as patients’ NHYA classification, length of time that they have had the disease, comorbidities and activities of daily living (ADL), and caregivers’ length of caregiving. The effectiveness of the interventions will be evaluated at the dyadic level and individual level for patients and their caregivers at baseline, immediately post-intervention, and at 3 months post-intervention.

All health outcome assessments will be tested using reliable and valid scales. The research assistants have been fully trained in outcome assessments to ensure quality and consistency; they will interview patients with CHF and their FCs to complete the online assessments (http://www.wenjuan.com/) via smartphone in a private office. Participants who discontinue the intervention will be encouraged to participate in the outcome assessments.

**Primary outcomes**

The primary outcomes are the patients’ HRQOL and their FCs’ HRQOL. The patients’ HRQOL will be comprehensively evaluated by the CHF-specific HRQOL scale utilizing the short form of the Kansas City Cardiomyopathy Questionnaire (KCCQ) (70) and by generic HRQOL measures using the Five-dimensional five-level EuroQol (EQ-5D-5L) (58), while the FCs’ HRQOL will be evaluated only by using the EQ-5D-5L(58).

**Specific CHF HRQOL**

The specific CHF-related HRQOL will be measured by employing the short form of the Kansas City Cardiomyopathy Questionnaire (KCCQ). The short form of the KCCQ consists of 12 items measured on a 5 or 7-point Likert scale with an overall score of between 0 to 100, with higher scores reflecting better HRQOL(70). This scale has demonstrated high responsiveness, test-retest reliability, and prognostic ability(70).

**Generic HRQOL**

The descriptive system of the Five-dimensional five-level EuroQol (EQ-5D-5L) will be used to evaluate generic HRQOL. The EQ-5D-5L is comprised of five items with five levels. The five items can result in a 5-digit number, which can be adopted to a single utility score by utilizing the Chinese scoring algorithm (71). The EQ-5D-5L has demonstrated good reliability with a Cronbach’s α of 0.857 and validity in the Chinese population (72).

**Secondary outcomes**

The secondary outcomes include measures at the dyadic level and individual level for patients and their caregivers.

**For patients and their family caregivers:**

**Perceived health status**

The EQ-5D-5L visual analog scale (EQ-5D-5L VAS) will be adopted to evaluate the individual’s perceived health status, which is comprised of a single global rating. Potential scores range from 0 to 100, with a higher score reflecting a better perceived health status (71). The reliability of the scale has been demonstrated with a Cronbach’s α of 0.83 among Chinese (73).

**Severity of anxiety**

The Generalized Anxiety Disorder Scale-7 (GAD-7) will be utilized to test the level of severity of anxiety. The GAD-7 consists of 74-point Likert items. The total score ranges from 0 to 21, with lower scores representing anxiety of less severity (74). The GAD-7 has shown good reliability with a Cronbach’s α of 0.91 and construct validity among Chinese (75).

**Severity of depressive symptoms**

The Patient Health Questionnaire (PHQ-9) will be employed to evaluate the level of severity of depressive symptoms. The PHQ-9 covers nine items measured on a four-point Likert scale. The total score ranges from 0 to 27, with lower scores representing less severe depression(76). The Cronbach’s α is 0.91, with good sensitivity and specificity among Chinese (75).

**Perceived quality of the relationship**

The short form of the Dyadic Adjustment Scale (DAS-7) will be adopted to examine the perceived quality of the relationship between the patients and their caregivers. The DAS-7 consists of 7 items measured on a 6 or 7-point Likert scale. The total score ranges from 0 to 36, with a higher score indicating a higher-quality relationship. It was used among CHF patients and caregivers and found to have acceptable Cronbach's α coefficients of 0.70-0.78 (77).

**Perspective-taking** **ability**

The most widely used scale to measure perspective-taking ability is the subscale on perspective-taking in the Interpersonal Reactivity Index (IRI) (78). The Chinese version of the IRI includes a 5-item subscale on perspective taking, measured on a 5-point Likert scale. The Cronbach's α coefficient of the perspective-taking subscale is 0.767 among Chinese (79).

- S**elf-compassion**

The short form of the Self-compassion scale (SCS–SF), which is comprised of 12 items, will be utilized to measure self-compassion (80). The Chinese version of SCS–SF demonstrated acceptable reliability, with a Cronbach’s α of 0.686, and validity among Chinese (81).

**Psychological flexibility**

The Comprehensive Assessment of Acceptance and Commitment Therapy Processes (CompACT) (82) will be utilized to evaluate the psychological flexibility of individuals. The Chinese version of CompACT consists of 18 items measured on a 7-point Likert scale. Scores range from 0 to 126, with a lower score representing a greater level of psychological flexibility. With a Cronbach's α of 0.87, the validity and reliability of CompACT have been demonstrated in a non-clinical sample in China (83).

**Patient-only outcomes:**

**Health service utilization**

The frequency of all course and cardiac-related readmissions and emergency department visits in any hospital will be assessed by self-reports.

**CHF self-care behavior**

The CHF self-care behavior of patients will be evaluated using the European Heart Failure Self-care Behavior Scale (EHFScBs) and self-reported smoking and alcohol drinking status. The ECHFScBs is comprised of 12 items measured on a 5-point Likert scale, and is used to evaluate CHF self-care behavior, with an emphasis on help-seeking and regimen-complying behavior(84). The possible total score ranges from 12 to 60, with a higher score indicating a lower level of self-care performance. The Chinese version of the ECHFScBs demonstrated good reliability, with a Cronbach’s α of 0.82, and validity among Chinese patients with CHF(85). As smoking and drinking behavior were excluded from the EHFScBs, patients will be asked to report their smoking and drinking status.

**Caregiver-only outcome:**

**Perceived caregiving burden**

The Zarit Caregiver Burden Interview (ZBI) will be used to evaluate feelings of caregiving burden (86). It consists of 22 statements measured on a 5-point Likert scale, with a total score ranging from 0 to 88. The Chinese version has shown good internal consistency (Cronbach's α 0.875) among Chinese caregivers and good validity(87).

# Procedure

Potential CHF patients will be initially identified by the staff nurses through reviewing electronic medical records. The staff nurses will contact the designated contact persons of the patients via telephone to obtain contact information of patients and their FCs. Then, both the patients and their FCs will be approached by phone and will be provided with an introduction to the study's purpose and procedure. If one member of patient-caregiver dyads is unable to participate, they will be required to phone back to inform the research team. If there are no response, the staff nurse will follow up with a phone call to provide further information on the study. Interested patient-caregiver dyads will be invited by the staff nurse to attend either through an online assessment via WeChat or through an in-person clinical visit to confirm eligibility.

After the eligibility of the patient and the patient’s FC is confirmed, the staff nurses at the Department of Cardiology will provide a detailed explanation of the study’s aim, methods, potential benefits, and risks, including all necessary elements of informed consent, either in the patient’s regular clinic or online via a WeChat video call. Once oral consent is given, the patient-FC dyads will receive a link to the information sheet via the WeChat platform. Verbally recorded informed consent will be sought from each CHF patient and their FC within one week after documents have been sent out over the smartphone.

Technical support before and during the sessions will be provided. Prior to the sessions, a video that shares step-by-step instructions on how to log onto VooV Meeting via a WeChat mini program will be provided to each dyad. Research assistants will conduct brief tutorials, either in person in the clinic or online via smartphone with dyads in the lead up to the first session to help them set up VooV meetings via the WeChat mini program. This is to ensure they are able to access group sessions and check their audio and video connections during the sessions. Throughout the program, research assistants will also provide timely online support on technical issues for the patient-FC dyads.

To promote recruitment and retention, the dyad will be given an incentive to undergo each assessment during the study, as compensation for their time; for example, each participating dyad will receive one set of CHF self-care materials for the baseline assessment, 50 Yuan RMB (≈6.97 USD) for each session, 50 Yuan RMB (≈6.97 USD) for the post-intervention assessments while 100 Yuan RMB (≈ 13.95 USD) for the 3-month follow-up assessment. The monetary incentives will be sent to the family via the WeChat platform. The material will be sent directly to the family by post.

A detailed schedule and timing of the evaluations are presented below in Table 2. T5 and T6 represent the time immediately post-intervention and at the 3-month follow-up point, respectively.

# Data management

A detailed database will be established to record the assessments and progress of each participant. Each participant will assign a unique identifier to preserve their confidentiality. This database will be password-protected and only accessible to authorized members of the research team. The researcher coordinator will monitor the data collection process, quality, and safety periodically. Any adverse events will be identified, documented, and handled with the caution. In cases of discrepancies, the research team including experts in cardiologist, nurses, and academia, will discuss and reach a consensus. Consultation with clinical trials specialists in the study hospital will be sought if required.

# Statistical analysis

All quantitative data collected from the participants will be numerical coded, and analyzed utilizing IBM SPSS 27. Baseline sociodemographic and clinical data will be used using medians, proportions, and counts for categorical data; while means, standard deviations (SDs), skewness, and kurtosis will be used for continuous variables for participants in two groups. Differences in the participants’ characteristics at baseline will be explored using an independent T-test or Mann-Whitney U test for continuous variables, and Chi-squared or Fisher’s Exact test for categorical variables. The primary outcome and secondary outcomes will be summarized using means with SDs for the ACT group and CE groups. Randomized testing at baseline between the study groups will be performed to identify potential covariates. The differences in these outcomes between the ACT group and the CE groups over time will be analyzed using generalized estimating equations (GEE) which account for missing data at random. If any covariance is detected in the baseline comparison, it will be adjusted in the analysis of the effects of the intervention. Subgroup analysis will be adopted for specific factors (e.g., intervention completion and non-completion). The level of statistical significance is predetermined at p<0.05 (two-tailed). The intention to treat principle will be applied by comparing the ACT group with CE groups as randomized despite adherence to the intervention. Cohen’s d will be calculated to estimate effect sizes of between-group comparisons.

**Ethical considerations**

Ethical approval for this study was given by the institutional review board (IRB) of the Hong Kong Polytechnic University (reference: HSEARS20210225006; 23-Apr-2021) and the Taihe hospital (Version 3; reference: 2022KS013; 3-Jun-2022;). Oral consent and verbally recorded informed consent will be obtained from each patient and family caregiver before a baseline assessment and randomization are conducted. Participation in the study is completely voluntary for CHF patient-caregiver dyads, and they are free to withdraw from the study at any point without consequences. Participants' identities and data, including information collected and video/audio records, will be kept confidential and anonymous to safeguard their privacy. Only authorized personnel will be allowed access to the data for analysis purposes.

**Dissemination**

The findings from this study will be published in referred journals. During the enrollment process, participants will be asked if they are interested in receiving findings or publications emanating from present study. The results will also be disseminated at international and national academic conferences and research seminars to promote knowledge sharing and guide healthcare initiatives for the broader CHF family.

REFERENCE

1. Ziaeian B, Fonarow GC. Epidemiology and aetiology of heart failure. Nature reviews Cardiology. 2016;13(6):368-78.

2. Ambrosy APMD, Fonarow GCMD, Butler JMDMPH, Chioncel OMD, Greene SJMD, Vaduganathan MMDMPH, et al. The global health and economic burden of hospitalizations for heart failure. Journal of the American College of Cardiology. 2014;63(12):1123-33.

3. Jackson JD, Cotton SE, Bruce Wirta S, Proenca CC, Zhang M, Lahoz R, et al. Burden of heart failure on patients from China: Results from a cross-sectional survey. Drug Design, Development and Therapy. 2018;12:1659-68.

4. Holden RJ, Schubert CC, Mickelson RS. The patient work system: an analysis of self-care performance barriers among elderly heart failure patients and their informal caregivers. Applied Ergonomics. 2015;47:133-50.

5. Cao X, Chair SY, Wang X, Chew HSJ, Cheng HY. Self-care in Chinese heart failure patients: gender-specific correlates. Heart & Lung. 2019;48(6):496-501.

6. Bosworth HB, Steinhauser KE, Orr M, Lindquist JH, Grambow SC, Oddone EZ. Congestive heart failure patients' perceptions of quality of life: the integration of physical and psychosocial factors. Aging & Mental health. 2004;8(1):83-91.

7. Giammanco MD, Gitto L. Coping, uncertainty and health-related quality of life as determinants of anxiety and depression on a sample of hospitalized cardiac patients in Southern Italy. Quality of Life Research. 2016;25(11):2941-56.

8. Seto E, Leonard KJ, Cafazzo JA, Masino C, Barnsley J, Ross H. Self-care and quality of life of heart failure patients at a multidisciplinary heart function clinic. Journal of Cardiovascular Nursing. 2011;26(5):377-85.

9. Cao X, Wang X-H, Wong EM, Chow CK, Chair SY. Type D personality negatively associated with self-care in Chinese heart failure patients. Journal of Geriatric Cardiology: JGC. 2016;13(5):401.

10. Sadeghiazar S, Mobasseri K, Gholizadeh L, Sarbakhsh P, Allahbakhshian A. Illness acceptance, medication adherence and the quality of life in patients with heart failure: A path analysis of a conceptual model. Applied Nursing Research. 2022;65:151583.

11. Grant JS, Graven LJ. Problems experienced by informal caregivers of individuals with heart failure: An integrative review. International Journal of Nursing Studies. 2018;80:41-66.

12. Clark AM, Wiens KS, Banner D, Kryworuchko J, Thirsk L, McLean L, et al. A systematic review of the main mechanisms of heart failure disease management interventions. Heart (British Cardiac Society). 2016;102(9):707-11.

13. Dirikkan F, Baysan Arabacı L, Mutlu E. The caregiver burden and the psychosocial adjustment of caregivers of cardiac failure patients. Türk kardiyoloji derneği arşivi 2018;46(8):692-701.

14. Hu X, Dolansky MA, Hu X, Zhang F, Qu M. Factors associated with the caregiver burden among family caregivers of patients with heart failure in southwest China. Nursing Health Sciences. 2016;18(1):105-12.

15. Pihl E, Jacobsson A, Fridlund B, Strömberg A, Måtensson J. Depression and health‐related quality of life in elderly patients suffering from heart failure and their spouses: a comparative study. European Journal of Heart Failure. 2005;7(4):583-9.

16. Scott LD. Caregiving and care receiving among a technologically dependent heart failure population. ANS Advances in nursing science. 2000;23(2):82-97.

17. Kim K-A, Park J-S, Seo K-W, Choi E-Y, Ahn J-A. Factors affecting the quality of life of family caregivers of patients with heart failure: A cross-sectional study. Medicine. 2022;101(35).

18. Chung ML, Moser DK, Lennie TA, Rayens MK. The effects of depressive symptoms and anxiety on quality of life in patients with heart failure and their spouses: Testing dyadic dynamics using Actor-Partner Interdependence Model. Journal of psychosomatic research. 2009;67(1):29-35.

19. Bidwell JT, Lyons KS, Lee CS. Caregiver well-being and patient outcomes in heart failure: A meta-analysis. Journal of Cardiovascular Nursing. 2017;32(4):372-82.

20. Retrum JH, Nowels CT, Bekelman DB. Patient and caregiver congruence: the importance of dyads in heart failure care. Journal of Cardiovascular Nursing. 2013;28(2):129-36.

21. Thomson P, Howie K, Leslie SJ, Angus NJ, Andreis F, Thomson R, et al. Evaluating emotional distress and health-related quality of life in patients with heart failure and their family caregivers: Testing dyadic dynamics using the Actor-Partner Interdependence Model. PLoS One. 2020;15(1):e0227129.

22. Ponikowski P, Voors AA, Anker SD, Bueno H, Cleland JGF, Coats AJS, et al. 2016 ESC Guidelines for the diagnosis and treatment of acute and chronic heart failure: the task force for the diagnosis and treatment of acute and chronic heart failure of the European Society of Cardiology (ESC) developed with the special contribution of the Heart Failure Association (HFA) of the ESC. European Journal of Heart Failure. 2016;37(27):2129-200.

23. Heart Failure Group of Chinese Society of Cardiology of Chinese Medical A, Chinese Heart Failure Association of Chinese Medical Doctor A, Editorial Board of Chinese Journal of C. Chinese guidelines for the diagnosis and treatment of heart failure 2018. Zhonghua XinXueGuanBing ZaZhi (in Chinese). 2018;46(10):760-89.

24. Hartmann M, Bäzner E, Wild B, Eisler I, Herzog W. Effects of interventions involving the family in the treatment of adult patients with chronic physical diseases: A meta-analysis. Psychother Psychosom. 2010;79(3):136-48.

25. Deek H, Hamilton S, Brown N, Inglis SC, Digiacomo M, Newton PJ, et al. Family-centred approaches to healthcare interventions in chronic diseases in adults: A quantitative systematic review. Journal of advanced nursing. 2016;72(5):968-79.

26. Buck HG, Stromberg A, Chung ML, Donovan KA, Harkness K, Howard AM, et al. A systematic review of heart failure dyadic self-care interventions focusing on intervention components, contexts, and outcomes. International Journal of Nursing Studies. 2018;77:232-42.

27. Srisuk N, Cameron J, Ski CF, Thompson DR. Randomized controlled trial of family-based education for patients with heart failure and their carers. Journal of advanced nursing. 2017;73(4):857-70.

28. Agren S, Evangelista LS, Hjelm C, Strömberg A, Ågren S, Evangelista LS, et al. Dyads affected by chronic heart failure: A randomized study evaluating effects of education and psychosocial support to patients with heart failure and their partners. Journal of Cardiac Failure. 2012;18(5):359-66.

29. Ågren S, Berg S, Svedjeholm R, Strömberg A. Psychoeducational support to post cardiac surgery heart failure patients and their partners—A randomised pilot study. Intensive and Critical Care Nursing. 2015;31(1):10-8.

30. Barriers and facilitators to self-care in chronic heart failure: a meta-synthesis of qualitative studies.

31. Graham CD, Simmons Z, Stuart SR, Rose MR. The potential of psychological interventions to improve quality of life and mood in muscle disorders. Muscle Nerve. 2015;52(1):131-6.

32. Hayes SC, Strosahl KD, Wilson KG. Acceptance and commitment therapy: An experiential approach to behavior change. New York, NY, US: Guilford Press; 1999. xvi, 304-xvi, p.

33. Graham CD, Gouick J, Krahe C, Gillanders D. A systematic review of the use of acceptance and commitment therapy (ACT) in chronic disease and long-term conditions. Clinical Psychology Review. 2016;46:46-58.

34. Han A, Yuen HK, Jenkins J. Acceptance and commitment therapy for family caregivers: A systematic review and meta-analysis. J Health Psychol. 2020;1-21:1359105320941217.

35. Mosher CE, Secinti E, Hirsh AT, Hanna N, Einhorn LH, Jalal SI, et al. Acceptance and commitment therapy for symptom interference in advanced lung cancer and caregiver distress: A pilot randomized trial. J Pain Symptom Manage. 2019;58(4):632-44.

36. Mosher CE, Secinti E, Wu W, Kashy DA, Kroenke K, Bricker JB, et al. Acceptance and commitment therapy for patient fatigue interference and caregiver burden in advanced gastrointestinal cancer: Results of a pilot randomized trial. Palliative Medicine. 2022;36(7):1104-17.

37. Lai FH, Yan EW, Yu KK, Tsui WS, Chan DT, Yee BK. The protective impact of telemedicine on persons with dementia and their caregivers during the COVID-19 pandemic. The American Journal of Geriatric Psychiatry. 2020;28(11):1175-84.

38. Brassington L, Ferreira NB, Yates S, Fearn J, Lanza P, Kemp K, et al. Better living with illness: A transdiagnostic acceptance and commitment therapy group intervention for chronic physical illness. Journal of Contextual Behavioral Science. 2016;5(4):208-14.

39. Bureau HPS. Shiyan statistical yearbook-2020. (in Chinese). Retrieved 25 September 2021. Available from: <https://web.archive.org/web/20210926013207/http://tjj.hubei.gov.cn/tjsj/sjkscx/tjnj/gsztj/sys/202101/P020210112435393051842.pdf>.

40. Callahan CM, Unverzagt FW, Hui SL, Perkins AJ, Hendrie HC. Six-item screener to identify cognitive impairment among potential subjects for clinical research. Medical Care. 2002:771-81.

41. Borson S, Scanlan JM, Chen P, Ganguli M. The Mini‐Cog as a screen for dementia: validation in a population‐based sample. Journal of the American Geriatrics Society. 2003;51(10):1451-4.

42. Samartzis L, Dimopoulos S, Tziongourou M, Nanas S. Effect of psychosocial interventions on quality of life in patients with chronic heart failure: a meta-analysis of randomized controlled trials. Journal of Cardiac Failure. 2013;19(2):125-34.

43. Gloster AT, Walder N, Levin ME, Twohig MP, Karekla M. The empirical status of acceptance and commitment therapy: A review of meta-analyses. Journal of Contextual Behavioral Science. 2020;18:181-92.

44. Ong CW, Lee EB, Twohig MP. A meta-analysis of dropout rates in acceptance and commitment therapy. Behaviour Research and Therapy. 2018;104:14-33.

45. Eifert GH, Forsyth JP. Acceptance and commitment therapy for anxiety disorders : A practitioner’s treatment guide to using mindfulness, acceptance, and values-based behavior change strategies: New Harbinger Publications.; 2005.

46. Luoma JB, Hayes SC, Walser RD. Learning ACT: An acceptance and commitment therapy skills-training manual for therapists. Oakland, CA, US: New Harbinger Publications; 2007. xi, 304-xi, p.

47. Tirch D, Schoendorff B, Hayes SC, Silberstein LR, Gilbert P. The ACT practitioner's guide to the science of compassion: Tools for fostering psychological flexibility: New Harbinger Publications; 2014.

48. McKay M. Acceptance and commitment therapy for interpersonal problems using mindfulness, acceptance, and schema awareness to change interpersonal behaviors. Lev A, Skeen M, ebrary I, editors. Oakland, Calif.: Oakland, Calif. : New Harbinger Publications; 2012.

49. Hayes SC, Smith S. Get out of your mind and into your life: The new acceptance and commitment therapy. Oakland: Oakland: New Harbinger Publications; 2005.

50. Lev A, McKay M. Acceptance and commitment therapy for couples: A clinician's guide to using mindfulness, values, and schema awareness to rebuild relationships. Oakland, CA, US: Context Press/New Harbinger Publications; 2017. vi, 251-vi, p.

51. Darrah Westrup JW. Learning ACT for group treatment:An acceptance and commitment therapy skills training manual for therapists.: Context Press.; 2017.

52. Bekelman DB, Nowels CT, Retrum JH, Allen LA, Shakar S, Hutt E, et al. Giving voice to patients' and family caregivers' needs in chronic heart failure: implications for palliative care programs. Journal of Palliative Medicine. 2011;14(12):1317-24.

53. Ahmad FS, Barg FK, Bowles KH, Alexander M, Goldberg LR, French B, et al. Comparing perspectives of patients, caregivers, and clinicians on heart failure management. Journal of Cardiac Failure. 2016;22(3):210-7.

54. McHorney CA, Mansukhani SG, Anatchkova M, Taylor N, Wirtz HS, Abbasi S, et al. The impact of heart failure on patients and caregivers: A qualitative study. PLoS One. 2021;16(3):e0248240.

55. Buck HG, Kitko L, Hupcey JE. Dyadic heart failure care types: Qualitative evidence for a novel typology. J Cardiovasc Nurs. 2013;28(6):E37-46.

56. Kim EY, Oh S, Son YJ. Caring experiences of family caregivers of patients with heart failure: A meta-ethnographic review of the past 10 years. European Journal of Cardiovascular Nursing. 2020;19(6):473-85.

57. Goodwin CL, Forman EM, Herbert JD, Butryn ML, Ledley GS. A pilot study examining the initial effectiveness of a brief acceptance-based behavior therapy for modifying diet and physical activity among cardiac patients. Behavior modification. 2012;36(2):199-217.

58. Majumdar S, Morris R. Brief group‐based acceptance and commitment therapy for stroke survivors. British Journal of Clinical Psychology. 2019;58(1):70-90.

59. Davis EL, Deane FP, Lyons GC, Barclay GD, Bourne J, Connolly V. Feasibility randomised controlled trial of a self-help acceptance and commitment therapy intervention for grief and psychological distress in carers of palliative care patients. Journal of Health Psychology. 2020;25(3):322‐39.

60. Losada A, Márquez-González M, Romero-Moreno R, Mausbach BT, López J, Fernández-Fernández V, et al. Cognitive-behavioral therapy (CBT) versus acceptance and commitment therapy (ACT) for dementia family caregivers with significant depressive symptoms: Results of a randomized clinical trial. Journal of Consulting And Clinical Psychology. 2015;83(4):760‐72.

61. Potter KJ, Golijana-Moghaddam N, Evangelou N, Mhizha-Murira JR, das Nair R. Self-help acceptance and commitment therapy for carers of people with multiple sclerosis: A feasibility randomised controlled trial. Journal of Clinical Psychology in Medical Settings. 2020.

62. Mosher CE, Secinti E, Hirsh AT, Hanna N, Einhorn LH, Jalal SI, et al. Acceptance and commitment therapy for symptom interference in advanced lung cancer and caregiver distress: a pilot randomized trial. Journal of Pain and Symptom Management. 2019;58(4):632-44.

63. Sandoz EK, Moyer DN, Armelie AP. Psychological flexibility as a framework for understanding and improving family reintegration following military deployment. Journal of Marital and Family Therapy. 2015;41(4):495-507.

64. Cano A, Corley AM, Clark SM, Martinez SC. A couple-based psychological treatment for chronic pain and relationship distress. Cognitive and Behavioral Practice. 2018;25(1):119-34.

65. Luoma JB, Hayes SC, Walser RD. Learning ACT: An acceptance & commitment therapy skills-training manual for therapists. Oakland, CA, US: New Harbinger Publications; 2007.

66. Artinian NT, Magnan M, Christian W, Lange MP. What do patients know about their heart failure? Applied Nursing Research. 2002;15(4):200-8.

67. Artinian NT, Magnan M, Sloan M, Lange MP. Self-care behaviors among patients with heart failure. Heart & Lung. 2002;31(3):161-72.

68. Van der Wal MH, Jaarsma T, Moser DK, van Veldhuisen DJ. Development and testing of the Dutch Heart Failure Knowledge Scale. European Journal of Cardiovascular Nursing. 2005;4(4):273-7.

69. Matsuoka S, Kato N, Kayane T, Yamada M, Koizumi M, Ikegame T, et al. Development and validation of a heart failure–specific health literacy scale. Journal of Cardiovascular Nursing. 2016;31(2):131-9.

70. Spertus JA, Jones PG. Development and validation of a short version of the Kansas City Cardiomyopathy Questionnaire. Circ Cardiovasc Qual Outcomes. 2015;8(5):469-76.

71. Herdman M, Gudex C, Lloyd A, Janssen M, Kind P, Parkin D, et al. Development and preliminary testing of the new five-level version of EQ-5D (EQ-5D-5L). Quality of Life Research. 2011;20(10):1727-36.

72. Xia J, Wu NW, Ma TP, Yu C, Li NX. Evaluation of reliability and validity of EQ-5D-5L based on residents in Southwest China. Journal of Sichuan University. 2020;51(5):691-4.

73. Lam CLK, Tse ETY, Wong CKH, Lam JSM, Chen SS, Bedford LE, et al. A pilot study on the validity and psychometric properties of the electronic EQ-5D-5L in routine clinical practice. Health and quality of life outcomes. 2021;19(1):266.

74. Spitzer RL, Kroenke K, Williams JB, Löwe B. A brief measure for assessing generalized anxiety disorder: The GAD-7. Archives of internal medicine. 2006;166(10):1092-7.

75. Zeng Q-Z, He Y-L, Liu H, Miao J-M, Chen J-X, Xu H-N, et al. Reliability and validity of Chinese version of the Generalized Anxiety Disorder 7-item (GAD-7) scale in screening anxiety disorders in outpatients from traditional Chinese internal department. . Chinese Mental Health Journal. 2013;27(3):163-8.

76. Chen S, Chiu H, Xu B, Ma Y, Jin T, Wu M, et al. Reliability and validity of the PHQ-9 for screening late-life depression in Chinese primary care. International Journal of Geriatric Psychiatry. 2010;25(11):1127-33.

77. Buck HG, Mogle J, Riegel B, McMillan S, Bakitas M. Exploring the relationship of patient and iInformal caregiver characteristics with heart failure self-care using the Actor-Partner Interdependence Model: Implications for outpatient palliative care. Journal of Palliative Medicine. 2015;18(12):1026-32.

78. Davis MHJJop. Measuring individual differences in empathy: Evidence for a multidimensional approach. Journal of Personality Social Psychology. 1983;44(1):113.

79. Rong X, Sun B, Huang X, Cai M, Li W. Reliabilities and validities of Chinese version of interpersonal reactivity index. . Chinese Journal of Clinical Psychology (in Chinese). 2010;18(2):158–60.

80. Raes F, Pommier E, Neff KD, Van Gucht D. Construction and factorial validation of a short form of the self‐compassion scale. Clinical Psychology. 2011;18(3):250-5.

81. Meng R, Yu Y, Chai S, Luo X, Gong B, Liu B, et al. Examining psychometric properties and measurement invariance of a Chinese version of the Self-Compassion Scale - Short Form (SCS-SF) in nursing students and medical workers. Psychology Research and Behavior Management. 2019;12:793-809.

82. Francis AW, Dawson DL, Golijani-Moghaddam N. Comprehensive assessment of acceptance and commitment therapy processes. Journal of Contextual Behavioral Science. 2016;5(3):134-45.

83. Yue C, Houyuan L, Shujuan W, Xiaoyu B, Zhuohong Z. Preliminary validation of a Chinese version of the comprehensive assessment of acceptance and commitment therapy processes. Current Psychology 2022.

84. Jaarsma T, Strömberg A, Mårtensson J, Dracup K. Development and testing of the European heart failure self‐care behaviour scale. European Journal of Heart Failure. 2003;5(3):363-70.

85. Yu DS, Lee DT, Thompson DR, Jaarsma T, Woo J, Leung EM. Psychometric properties of the Chinese version of the European heart failure self-care behaviour scale. Internaitonal Journal of Nursing Studies. 2011;48(4):458-67.

86. Zarit SH, Reever KE, Bach-Peterson J. Relatives of the impaired elderly: correlates of feelings of burden. Gerontologist. 1980;20(6):649-55.

87. Lu L, Wang L, Yang X, Feng Q. Zarit Caregiver Burden Interview: Development, reliability and validity of the Chinese version. Psychiatry Clinical Neuroscience. 2009;63(6):730-4.
