# Supplementary material for: Effectiveness of a videoconferencing group-based dyad acceptance and commitment therapy on the quality of life of chronic heart failure patients and their family caregivers: A study protocol for a randomized controlled trial
Source: PLoS One. 2024 Apr 18;19(4):e0298178. doi: 10.1371/journal.pone.0298178 (PMC11025806; doi:10.1371/journal.pone.0298178)
Supplement: Attachment — Submitted filename: Comments.docx [file pone.0298178.s003.docx]

Thanks for giving the opportunity to review the paper. The authors report a study protocol of ACT in chronic heart failure patients and their family caregivers. The topic is interesting and relevant to the journal, however, the manuscript has a number of issues that must be addressed before it can be considered further for publication. Specific comments are listed below.

Title:

1. Will the authors consider clarifying the primary outcome of the study in the title?

Introduction:

1. Lines 41-42. Could this sentence be more specific? What do patients anxious/depressive about? Any specific uncertainty experiences? Please also add citations for them.
2. It is suggested to add an optional definition for HRQOL.
3. Lines 51-52, how the psychological states are linked to HRQOL. Please specify their relationships.
4. As HRQOL is the primary outcome of the study, it could better to highlight the HRQOL of patients and caregivers and the impacts in an independent paragraph.
5. Please make sure that a thorough literature review of non-pharmacological interventions on dyads with chronic illness has been conducted with references citations. Please also be specific in summarising the limitations of current interventions on your target population. E.g. lines 66-68, why CBT and motivation enhancement are not sufficient to promote CHF self-management.
6. Line: it is unclear on ‘circumstances of context’
7. More than two studies have been conducted amongst patients with advanced cancer and their caregivers. Please make sure a thorough literature review was conducted again. Besides, why only review ACT studies on advanced cancer dyads? Any previous studies of ACT on other chronic illnesses that could be learned about?
8. It is suggested to highlight the necessity/significance to conduct an online-based ACT in your target population.
9. Line 83, ‘previous studies’ in which population?

Methods:

1. Lines 128-129, how 14 hours is identified? Any references?
2. Lines 147-148, ‘The authors report on a meta-analysis of ACT on psychological and quality of life outcomes in patients with advanced cancer.’ In this case, how could the authors ensure the study consistent with the planned frequency and duration of the intervention?
3. Line 186, please specify the ‘registered nurse’ from the author list, the principal investigator?
4. In the intervention section, the author said each session will be led by a registered nurse. In the intervention group, two registered nurse were described. Please be specific.
5. Lines 212-213, could authors specify the details of the revisions on Version 1 protocol?
6. Lines 243-244, as the second facilitator has a bachelor’s degree and only received two-day ACT training, which is different from the first facilitator who is a doctoral student with 28-day training, how could the author make sure this facilitator is able to independently deliver the interventions to participants?
7. Why patient’s HRQOL will be evaluated by two different scales?
8. Has the protocol been registered? If Yes, please provide the registered number.
