# Supplementary material for: Effectiveness of a videoconferencing group-based dyad acceptance and commitment therapy on the quality of life of chronic heart failure patients and their family caregivers: A study protocol for a randomized controlled trial
Source: PLoS One. 2024 Apr 18;19(4):e0298178. doi: 10.1371/journal.pone.0298178 (PMC11025806; doi:10.1371/journal.pone.0298178)
Supplement: Attachment — Submitted filename: Response to Reviewers-2.docx [file pone.0298178.s005.docx]

Below is a summary of our response to the comments:

| **Comments** | **Response (highlighted the revision made in the manuscript)** |
| --- | --- |
| Reviewer #1:  The paper has been improved; some minor comments are as below: 1. The authors stated, ‘These emotional and behavioral responses exhibited by each member within a dyad can have a reciprocal influence on the other (40, 41), resulting in a decrease in the quality of their relationship (42), poorer physical functioning (43), and reduced QOL (36, 44) for both patients and their FCs.’ Please be more specific on how emotional and behavioral responses of each member influence the other. | Thank you for your comments. We have added the statement regarding the emotional and behavioral responses within the dyad as below.  However, engaging in CHF self-care activities, such as taking note of food items or seeking professional help, can have the effect of causing the individual to establish a psychological connection to the tangible and potentially distressing consequences associated with CHF, thereby evoking thoughts about the illness and eliciting reactions to its potential dangers. For instance, CHF patients may relate their interactions with healthcare providers to their "sick identity". This may lead them to choose to avoid seeking professional help as a way to deny their CHF disease and cope with feelings of abnormality (21). FCs often dedicate to providing competent and timely care (22). Many FCs respond with unconscious excessive vigilance in ensuring the well-being and comfort of their patients, driven by their fear of potential future losses (23, 24). This heightened vigilance may persist even when the patient’s health is relatively stable and care demands are low (25, 26). |
| 2. Please consider shorten the length of the introduction, as this part is a bit long. | Thank you for your suggestion. We have shortened the introduction to 129 lines. |
| 3. Language editing is needed before resubmission. | Thank you for your suggestion. The paper has been edited by a professional editor |
